# Association of Adverse Childhood Experiences With Poor Neuropsychiatric Health and Dementia Among Former Professional US Football Players

**DOI:** 10.1001/jamanetworkopen.2022.3299

**Published:** 2022-03-22

**Authors:** Andrea L. Roberts, Ross Zafonte, Lori B. Chibnik, Aaron Baggish, Herman Taylor, Jillian Baker, Alicia J. Whittington, Marc G. Weisskopf

**Affiliations:** 1 Department of Environmental Health, Harvard T. H. Chan School of Public Health, Boston, Massachusetts; 2 Football Players Health Study at Harvard University, Harvard Medical School, Boston, Massachusetts; 3 Department of Neurology, Massachusetts General Hospital, Boston; 4 Department of Epidemiology, Harvard T. H. Chan School of Public Health, Boston, Massachusetts

## Abstract

**Importance:**

Childhood adversities, including neglect, abuse, and other indicators of family dysfunction, are associated in adulthood with risk factors for poor cognitive and mental health. However, the extent to which these experiences are associated with adulthood cognition-related quality of life and risk for dementia is unknown.

**Objective:**

To determine the association of 10 adverse childhood experiences (ACEs) with neuropsychiatric outcomes among former National Football League (NFL) players.

**Design, Setting, and Participants:**

This cross-sectional analysis used data from the Football Player’s Health Study at Harvard University, an ongoing longitudinal cohort study from January 30, 2015, to November 19, 2021, of former NFL players.

**Exposures:**

Ten ACEs were assessed using the Adverse Childhood Experiences Questionnaire.

**Main Outcomes and Measures:**

Dementia symptoms were assessed using the AD8: The Washington University Dementia Screening Test; cognition-related quality of life was assessed with the short form of the Quality of Life in Neurological Disorders; depression was assessed with the Patient Health Questionnaire–9; anxiety was assessed with the Generalized Anxiety Disorder–7; and pain intensity and pain interference in daily life were assessed with the Brief Pain Inventory. Risk ratios (RRs) assessing the association between ACEs and neuropsychiatric outcomes were estimated using generalized estimating equations, adjusted for age, race, and childhood socioeconomic status, and further adjusted for playing position, concussions incurred during football play, and number of seasons played in the NFL.

**Results:**

A total of 1755 men (mean [SD] age, 57.2 [13.5] years) who were former professional football players were included in the analysis. Five hundred twenty players (29.6%) identified as Black, 1160 (66.1%) identified as White, and 75 (4.3%) identified as other race or ethnicity. Players with 4 or more ACEs were at 48% greater risk of a positive screen for dementia (RR, 1.48 [95% CI, 1.22-1.79]), and at significantly greater risk of every other neuropsychiatric outcome except anxiety (RR range, 1.62 [95% CI, 1.09-2.39] to 1.74 [95% CI, 1.27-2.40]) compared with players with no ACEs. Further adjustment for concussions incurred during playing years attenuated these associations, although some were still significant (adjusted RR range, 1.32 [95% CI, 1.10-1.58] to 1.56 [95% CI, 1.15-2.11]). ACEs were also associated with concussion symptoms; players with 4 or more ACEs had a 60% increased risk of being in the top quartile of concussion symptoms (RR, 1.60; 95% CI, 1.12-2.28) compared with players with no ACEs.

**Conclusions and Relevance:**

These findings suggest that ACEs may be associated with dementia symptoms among former NFL players. Moreover, ACEs should be investigated among professional football players and other populations as a prospective indicator of persons at high risk of concussion. These findings further suggest that treatment of psychological trauma in addition to treatment of physical injury may improve neuropsychiatric health in former NFL players.

## Key Points

**Question:**

Are adverse childhood experiences (ACEs) associated with dementia symptoms and poor neuropsychiatric health in former professional football players?

**Findings:**

In this cross-sectional study of 1755 former professional US football players, 10 ACEs, primarily indicators of family dysfunction, were associated with a positive dementia screening result. Players with at least 4 ACEs were 48% more likely to have a positive finding on a dementia screen and were at greater risk of poor cognition-related quality of life, pain, and depression compared with players with no ACEs.

**Meaning:**

These findings suggest that childhood family dysfunction may be a risk factor for dementia symptoms and poor neuropsychiatric health in adulthood in former professional football players.

## Teaser

This cross-sectional study investigates the association of adverse childhood experiences with poor neuropsychiatric health and dementia among former National Football League players and whether football-related concussions accounted for the association.

## Introduction

Cognitive and mental health are major concerns for former US football players owing to established associations of concussion exposure with problems involving thinking, memory, and mood. Depression, anxiety, and pain can follow concussions^1^ and may contribute to cognitive problems in former football players.^2,3^ In addition to physical trauma to the brain, psychological trauma has been linked to risk factors for poor cognitive function. In particular, childhood abuse and other indicators of family dysfunction, termed *adverse childhood experiences* (ACEs), are associated with depression, anxiety, and pain in adulthood.^4,5^ ACEs have been reported to affect learning and memory in childhood,^6^ and these effects may persist into adulthood.^7,8,9^ However, only 1 study^10^ has examined the association of ACEs with dementia, and none have examined cognition-related quality of life.

In addition, adults who experienced childhood neglect and abuse, compared with those who did not, are more likely to have low self-esteem,^11,12^ engage in risk-taking behaviors,^13,14,15^ be more aggressive,^16,17^ and harm themselves.^18^ Thus, men who have been exposed to ACEs may be more likely than nonexposed men to play football with more aggression and disregard for their own safety, resulting in a higher risk of concussion.

We investigated the association between ACEs and neuropsychiatric health, including positive dementia screening results, cognition-related quality of life, depression, anxiety, and pain, cross-sectionally in a cohort of former professional football players. We further examined whether ACEs were associated with football-related concussion symptoms during playing years and if so, whether concussions might account for a possible association between ACEs and poor neuropsychiatric health. Finally, we examined whether playing position and number of years of play might account for this association. We hypothesized that ACEs would be associated with poor neuropsychiatric health and that concussions and playing position would account for part of this association.

## Methods

### Sample

The Football Player’s Health Study at Harvard University is an ongoing longitudinal cohort of former National Football League (NFL) players. All former players who had signed with an NFL team since 1960 were eligible, with enrollment ongoing. From January 30, 2015, to December 31, 2018, 3833 men who were former football players responded to the first questionnaire. Players were invited to complete a second questionnaire 2 or more years later, from January 1, 2019, to November 19, 2021.^19^ As of November 2021, 1770 players had responded to the wave 2 questionnaire (46.2% of wave 1 respondents). Of these, 1755 responded to ACE questions and constituted our study sample. This cross-sectional study was approved by the Harvard School of Public Health institutional review board and followed the Strengthening the Reporting of Observational Studies in Epidemiology (STROBE) reporting guideline. Participants provided written informed consent.

### Measures

#### Adverse Childhood Experiences (Wave 2)

We queried players about 10 ACEs using the Adverse Childhood Experiences Questionnaire.^20,21^ The questionnaire has been validated against the Adult Attachment Interview,^22^ has good to excellent test-retest reliability,^23^ and has been associated in a dose-dependent manner with adulthood depression, obesity, smoking, and premature death.^24,25,26,27,28^ The questionnaire is available publically.^29^

The following experiences in the first 18 years of life were queried with yes or no response options: physical abuse; sexual abuse by an adult or person 5 or more years older (ie, sexual touching or attempted or completed intercourse); emotional abuse (eg, “Did a parent or adult in the household often… swear at… or humiliate you?”); emotional neglect (eg, “no one in your family loved you or thought you… were special?”); physical neglect (eg, did not have enough to eat); intimate partner violence; household member imprisoned; household member with mental illness; household member who abused alcohol or drugs; and parents ever separated or divorced. Following standard protocol,^5,30^ we summed the ACEs and created categories of none, 1, 2, 3, and 4 or more.

#### Neuropsychiatric Outcomes (Wave 2)

A positive dementia screening result was ascertained with the AD8: The Washington University Dementia Screening Test (hereinafter referred to as the AD8).^31^ Players were asked about changes in memory and thinking during the last several years (eg, trouble remembering appointments) with the following response options: yes, a change; no, no change; or N/A [not applicable], don’t know. The number of yes responses were summed, with 2 or more indicating probable dementia. In a study comparing the AD8 with the Clinical Dementia Rating (CDR) system using informant report, a cutoff of 2 distinguished a person with very mild dementia (CDR score, 0.5) from persons without dementia (CDR score, 0) with good to excellent psychometrics.^31^ The AD8 has also been validated using self-report with good psychometrics.^32^

Cognition-related quality of life was measured by the 8-item short form of the Quality of Life in Neurological Disorders.^33,34^ Past 7-day cognitive difficulties were queried. Based on published guidelines, we created an indicator of moderate or severe impairment using a score of 40 or less, which corresponds to at least 1 SD below the US population mean.^33,35^

Depressive symptoms were measured with the Patient Health Questionnaire–9.^36,37^ For each of 9 symptoms, respondents indicated whether the symptom bothered them not at all (0) to nearly every day (3) during the previous 2 weeks. Responses were summed and dichotomized at 15 or greater to indicate probable moderate or severe depression, based on a validation study with good psychometrics.^37^ Past 2-week anxiety symptoms (eg, feeling nervous, anxious, or on edge) were queried using the Generalized Anxiety Disorder–7,^38^ with the same response options and scoring as depressive symptoms. The Generalized Anxiety Disorder–7 score was dichotomized at 10 or greater to indicate probable moderate or severe anxiety, as determined by comparison with a structured psychiatric interview.^38^

The Brief Pain Inventory^39,40^ was used to measure the past 24-hour pain, querying pain severity at its worst, its least, its average, and right now. Each item was rated as none (0) through as bad as you can imagine (10). Eight items addressed the past 24-hour pain interference in daily life with ratings of does not interfere (0) through completely interferes (10). Although pain is subjective, generalizations of these descriptive levels are used in clinical practice. Mild pain does not seriously impair or distract. Moderate pain is difficult to ignore and disruptive to mood, activities, and sleep. Severe pain dominates the senses and prohibits most activities. Each scale of the Brief Pain Inventory was dichotomized according to validation studies, with scores of 5 or greater indicating moderate or severe intensity or interference in daily life.^41,42^

#### Football Exposures (Wave 1)

Because diagnosed concussions are known to be a poor measure of concussion history owing to nondisclosure or nontreatment,^43^ we used self-report of concussion symptoms during playing years as a proxy for concussion history. Players were asked “While playing or practicing football, did you experience a blow to the head, neck, or upper body followed by headaches, nausea, dizziness, loss of consciousness, memory problems, disorientation, confusion, seizure, visual problems, and feeling unsteady on your feet?” Response options for each symptom were no, once, 2 to 5 times, 6 to 10 times, or 11 or more times. These were coded 0, 1, 3.5, 8, and 13, respectively; the mean across all 10 items was calculated, and the resulting score was divided into quartiles.^19^ Players were asked to indicate the positions they most often played professionally. Responses were combined into 3 groups based on risk of concussion symptoms at time of football injury in our sample^19^: (1) quarterback, kicker, or punter; (2) wide receiver, defensive back, lineman, or tight end; and (3) running back, linebacker, or special teams. Seasons of professional football were self-reported.

#### Covariates

Childhood socioeconomic status (SES) was queried with 5 constructs: maternal and paternal job type, maternal and paternal educational level, and food insecurity. Parents’ work during respondent’s childhood was queried, with 10 response options covering a range of job types. Occupational status was then coded as a categorical variable for each parent separately as relatively unskilled (eg, laborer, janitor, guard), skilled (eg, military, mechanic, farmer, machine operator), or white collar (eg, professional, business owner, executive). Educational attainment of each parent was queried and coded separately as a categorical variable with 5 levels: less than high school graduate, high school graduate, some college, college graduate, and more than college. Frequency of food insecurity while growing up was measured with 2 questions: “I worried whether our food would run out before we got money to buy more” and “The food my family bought just didn’t last and we didn’t have money to get more.”^44^ Response options were never, rarely, sometimes, and often. Food insecurity was coded as a categorical variable based on the most frequent of these circumstances (eg, if the response to either question was “often,” then the respondent was considered often food insecure).

Racial identity was queried because it has been associated with health outcomes. Players selected 1 or more from the following options: American Indian or Alaska Native, Asian, Black, Native Hawaiian or Pacific Islander, White, or other. Racial identity was coded as Black, White, or other. Birth year was self-reported.

### Statistical Analysis

We calculated the prevalence of demographic characteristics and football exposures by the number of ACEs. To estimate the association of the number of ACEs with our outcomes after adjusting for age, race, and childhood SES, we calculated risk ratios (RRs) using generalized estimating equations with a log link and a Poisson distribution.^45^ To examine whether the prevalence of concussion symptoms at the time of football injury might mediate an association of ACEs with outcomes, we further adjusted for concussion symptoms in quartiles. Finally, to determine whether years of play and playing position might account for associations, we fit a third model further adjusted for these factors.

To examine whether ACEs were associated with concussion symptoms during playing years, we modeled risk for being in the highest quartile of concussion symptoms as the dependent variable, with ACE score as the independent variable, in models adjusted for age, race and ethnicity, and childhood SES and further adjusted for playing position and years of play, using generalized estimating equations with a log link and a Poisson distribution. To reduce possible bias associated with current depression and anxiety on recall of concussion symptoms, we examined the association of ACEs with concussion risk restricted to players without depression or anxiety at the time of the wave 2 questionnaire. Analyses were conducted in SAS, version 9.4 (SAS Institute Inc), with 2-sided *P* < .05 considered statistically significant.

## Results

Among the 1755 men included in the analysis, all of whom were former professional football players, the mean (SD) age was 57.2 (13.5) years (range, 28-92 years). The median time from wave 1 to wave 2 was 4 (IQR, 4-5) years. Nearly one-third of the players identified as Black (520 [29.6%]), two-thirds identified as White (1160 [66.1%]), and the remainder (75 [4.3%]) identified as American Indian or Alaska Native, Asian, Native Hawaiian or Pacific Islander, or other race or ethnicity. Compared with players who did not participate in wave 2, players who participated were slightly older (mean [SD] age, 53.1 [13.5] vs 51.2 [15.0] years), more likely to identify as White (1160 [66.1%] vs 1217 [52.1%]), and less likely to be in the top quartile of concussion symptoms (365 [20.8%] vs 652 [27.6%]). The prevalence of ACEs ranged from 767 participants (43.7%) with no ACEs to 208 (11.9%) with 4 or more ACEs.

Divorce (527 [30.0%]), living with a family member with an alcohol use problem or substance use (406 [23.1%]), and physical abuse (363 [20.7%]) were the most common ACEs (Table 1). ACEs were associated with childhood SES. For example, food insecurity was experienced by 32 of 767 players with no ACEs (4.2%) and 93 of 208 players with 4 or more ACEs (44.7%). The number of ACEs was associated with race and ethnicity, with White identity being more prevalent (598 of 767 [78.0%] with 0 ACEs vs 89 of 208 [42.8%] with ≥4 ACEs) and Black identity being less prevalent (137 of 767 [17.9%] with 0 ACEs vs 107 of 208 [51.4%] with ≥4 ACEs) among players with fewer vs more ACEs. The number of ACEs was also associated with playing position, with the positions of quarterback, kicker, or punter being more prevalent among players with no ACEs (81 of 767 [10.6%]) vs 4 or more ACEs (8 of 208 [3.8%]). Concussion symptoms were associated in dose-dependent fashion with ACEs. Among the 767 players with no ACEs, 123 (16.0%) were in the top quartile of concussion symptoms, whereas among the 208 players with 4 or more ACEs, 66 (31.7%) were in the top quartile (Table 2).

**Table 1.** Prevalence of ACEs and Neuropsychiatric Outcomes in the Football Players Health Study, 2018-2021

Exposures and outcomes	Participants, No. (%) (N = 1755)
ACE exposures	
Emotional abuse	337 (19.2)
Physical abuse	363 (20.7)
Sexual abuse	111 (6.3)
Emotional neglect	117 (6.7)
Physical neglect	82 (4.7)
Divorce or separation	527 (30.0)
Intimate partner violence toward mother	147 (8.4)
Household member with alcohol use problem or substance use	406 (23.1)
Household member with mental illness or suicidal	169 (9.6)
Household member imprisoned	134 (7.6)
Neuropsychiatric outcomes	
Positive dementia screening result	752 (42.8)
Poor cognition-related quality of life	656 (37.4)
Probable moderate or severe depression	291 (16.6)
Probable moderate or severe anxiety	194 (11.1)
High pain severity	556 (31.7)
High pain interference	421 (24.0)

Abbreviation: ACE, adverse childhood experience.

**Table 2.** Association of Childhood Adversities With NFL Position, Years of Play, and Concussion Symptoms, Football Players Health Study, 2018-2021

Characteristic	No. of participants (N = 1755)	No. of ACEs^a^				
		0 (n = 767)	1 (n = 400)	2 (n = 245)	3 (n = 135)	≥4 (n = 208)
Age at questionnaire, mean (SD), y	1755	59.4 (13.8)	55.7 (13.9)	56.3 (12.2)	56.3 (12.4)	54.3 (12.5)
Racial identity						
Black	520	137 (17.9)	129 (32.3)	95 (38.8)	52 (38.5)	107 (51.4)
White	1160	598 (78.0)	256 (66.3)	141 (57.5)	76 (56.3)	89 (42.8)
Other^b^	75	32 (4.1)	15 (37.5)	9 (3.7)	7 (5.2)	12 (5.8)
Football exposures						
Time in NFL, mean (SD), y	1755	6.7 (4.0)	6.4 (3.7)	6.7 (3.5)	6.9 (4.1)	6.5 (3.9)
Playing position						
Quarterback, kicker, or punter	151	81 (10.6)	34 (8.5)	16 (6.5)	12 (8.9)	8 (3.8)
Wide receiver, defensive back, lineman, or tight end	889	391 (51.0)	201 (50.3)	116 (47.3)	65 (48.1)	116 (55.8)
Running back, linebacker, or special teams	715	295 (38.5)	165 (41.3)	113 (46.1)	58 (43.0)	84 (40.4)
Concussion symptoms during playing years, highest quartile	365	123 (16.0)	83 (20.7)	53 (21.6)	40 (29.6)	66 (31.7)
Childhood SES						
Parental occupation, unskilled	171	54 (7.0)	33 (8.3)	27 (11.0)	17 (12.6)	40 (19.2)
Parental education, less than high school	172	69 (9.0)	32 (8.0)	25 (10.2)	16 (11.9)	30 (14.4)
Food insecurity, sometimes or often	217	32 (4.2)	29 (7.3)	38 (15.5)	25 (18.5)	93 (44.7)

Abbreviations: ACE, adverse childhood experience; NFL, National Football League; SES, socioeconomic status.

^a^ Unless otherwise indicated, data are expressed as number (%) of participants. Percentages have been rounded and may not total 100.

^b^ Includes American Indian or Alaska Native, Asian, Native Hawaiian or Pacific Islander, or other race or ethnicity.

Correlations for individual ACEs were calculated, with the largest point estimates between emotional neglect and abuse (*r* = 1.00), emotional abuse and domestic violence (*r* = 0.35), physical abuse and domestic violence (*r* = 0.35), and emotional and physical neglect (*r* = 0.31) (eTable 1 in the Supplement). Positive dementia screening results (752 [42.8%]) and poor cognition-related quality of life (656 [37.4%]) were highly prevalent in our sample (Table 1). Correlations for neuropsychiatric outcomes were calculated. Positive dementia screening results and cognition-related quality of life (*r* = 0.67), probable depression and probable anxiety (*r* = 0.59), and pain severity and pain interference in daily activities (*r* = 0.57) were most highly correlated (eTable 2 in the Supplement).

In models adjusted for age, race and ethnicity, and childhood SES, ACEs were associated in an increasing fashion with each outcome, although associations with anxiety did not reach statistical significance. Experiencing 4 or more ACEs was associated with more than 40% greater risk of each outcome compared with experiencing no ACEs. Of note, risk of a positive dementia screening result was 48% higher in players with at least 4 ACEs compared with those with no ACEs (RR, 1.48 [95% CI, 1.22-1.79]) (Figure). The largest point estimates were for ACEs and pain interference in daily life (74% more likely for those with ≥4 ACEs [RR, 1.74 (95% CI, 1.27-2.40)]), poor cognition-related quality of life (65% more likely for those with ≥4 ACEs [RR, 1.65 (95% CI, 1.33-2.04)]), and probable depression (62% more likely for those with ≥4 ACEs [RR, 1.62 (95% CI, 1.09-2.39)]). Further adjustment for concussions attenuated all associations (adjusted RR range, 1.21 [95% CI, 0.74-1.96] to 1.56 [95% CI, 1.15-2.11]), although associations with dementia (RR, 1.32 [95% CI, 1.10-1.58]), poor cognition-related QOL (RR, 1.47 [95% CI, 1.21-1.79]), and both pain intensity (RR, 1.37 [95% CI, 1.07-1.74]) and interference (RR, 1.56 [95% CI, 1.15-2.11]) remained statistically significant (eTable 3 in the Supplement). The results were nearly identical in models additionally adjusted for seasons of professional play and playing position.

**Figure.**
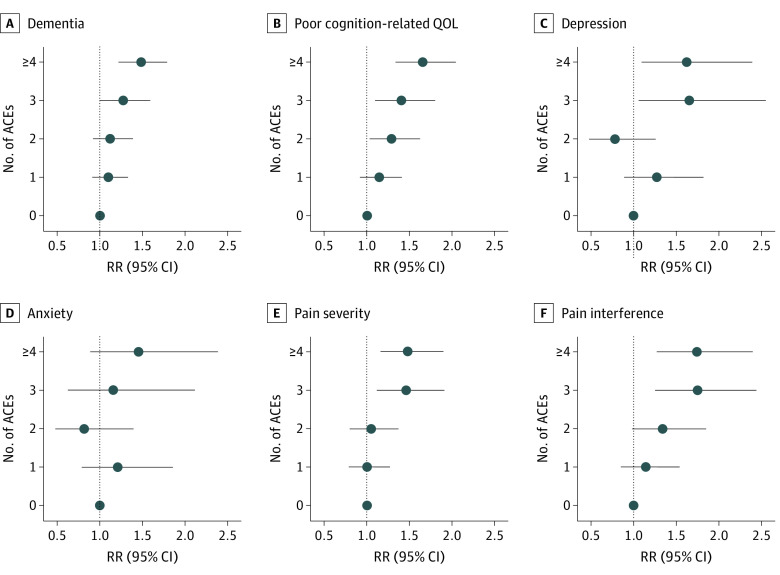
Adverse Childhood Experiences and 6 Neuropsychiatric Health Outcomes Data are from 1755 participants in the 2018-2020 Football Players Health Study. ACE indicates adverse childhood experience; QOL, quality of life; RR, risk ratio.

ACE score was associated with a 60% increased risk of concussion symptoms in models adjusted for age, race and ethnicity, and childhood SES (≥4 ACEs [RR, 1.60 (95% CI, 1.12-2.28)]) (Table 3). Further adjustment for playing position and years of play did not notably alter these estimates. Associations were similar in analyses restricted to players without depression or anxiety.

**Table 3.** ACEs and Risk of Being in the Top Quartile of Concussion Symptoms at the Time of Football Injury, Football Players Health Study, 2018-2021

	No. of ACEs^a^					*P* value, test of trend
	None	1	2	3	≥4	
Entire sample, No.	767	400	245	135	208	NA
Base model^b^	1 [Reference]	1.23 (0.91-1.67)	1.15 (0.79-1.65)	1.62 (1.11-2.36)	1.60 (1.12-2.28)	<.001
Further adjusted for position and seasons in NFL	1 [Reference]	1.20 (0.89-1.63)	1.10 (0.77-1.57)	1.58 (1.09-2.30)	1.57 (1.10-2.25)	.007
Players without probable depression or anxiety only, No.	661	325	202	103	132	NA
Base model^b^	1 [Reference]	1.31 (0.99-1.92)	1.30 (0.83-2.05)	1.26 (0.72-2.21)	1.61 (0.99-2.63)	.07

Abbreviations: ACE, adverse childhood experiences; NA, not applicable; NFL, National Football League.

^a^ Includes 1755 participants. Unless otherwise indicated, data are expressed as risk ratio (95% CI).

^b^ Adjusted for age, racial identity, and childhood socioeconomic status, measured by parents’ occupation and educational level and frequency of food insecurity in childhood.

## Discussion

We found an association between childhood adversities and poor neuropsychiatric health. Notably, we found that players who had experienced at least 4 ACEs were 48% more likely to have a positive dementia screening result and 65% more likely to have poor cognition-related quality of life than those who had experienced no ACEs after adjustment for age, racial identity, and childhood SES. To our knowledge, only 1 prior study^46^ has examined the association of childhood abuse and family dysfunction with dementia. Aboriginal and Torres Strait Islander Australians (n = 296) reported childhood maltreatment on the Childhood Trauma Questionnaire.^46^ A 1-SD increase in the maltreatment score was associated with 70% increased odds of dementia and 77% increased odds of Alzheimer disease.^10^ Although many studies have indicated that abuse impairs cognitive function in childhood,^47,48^ only 1 study^9^ has examined the association of abuse with later-life cognitive function, finding lower cognitive function at 49 to 69 years of age in women who had been exposed to abuse vs those who had not.

The prevalence of ACEs in our sample was similar to that in a representative sample of 53 998 men from 10 US states^27^ (no ACEs, 767 [43.7%] in the Football Player’s Health Study vs 40.6% in the representative sample; ≥4 ACEs, 208 [11.9%] in the Football Player’s Health Study vs 13.3% in the representative sample). Our findings that ACEs were associated with depression and severe and disabling pain are similar to findings in nonathletes^49,50,51^ and suggest that mood disorders and pain may be important factors in the association of ACEs with dementia and cognition-related quality of life among former football players. Longitudinal studies of players that begin follow-up at the start of their NFL careers could help determine the sequencing of depression, anxiety, pain, and cognitive impairment, to best design interventions. In addition, prior studies have indicated that persons exposed to childhood abuse have an elevated prevalence of nearly every risk factor for dementia, such as smoking, hypertension, and type 2 diabetes,^52,53,54,55,56,57^ and these factors may also play a role in the association of ACEs with cognitive outcomes in former NFL players. Early life adversity, subsequent health risk factors, and pathology linked to repeated brain trauma may together produce a complex clinical phenotype in former football players and others with these exposures.

We found an association of ACEs with concussion symptoms during professional football careers, which statistically accounted for some of the elevated risk of poor neuropsychiatric health. One possible explanation is that players who have been exposed to ACEs may be more likely than nonexposed players to have a more aggressive, risk-taking playing style and possibly disregard their own safety owing to low self-esteem,^11,12^ risk-taking behaviors,^13,14,15^ and aggressiveness resulting from their exposure.^16,17^ Thus, there may be differences in how players approach the game that affect their concussion exposure, raising the possibility that players—particularly those with a history of multiple ACEs—could be taught safer approaches.^58,59^

A second possibility is that players who experienced ACEs experienced worse symptoms after a concussion, which may explain why they scored higher on our concussion symptom score. Presence of depression, anxiety, and psychosocial stressors immediately after concussion and prior mental and behavioral health problems are known to be associated with postconcussion syndrome,^60,61,62^ defined as concussion symptoms that last beyond the expected recovery period.^60,63^ A third possibility is that players who experienced ACEs had biased recall of their concussion symptoms, leading to reporting more symptoms than they experienced. Conversely, players with few or no ACEs may have been biased toward recalling symptoms as being less severe than they were. However, associations of ACEs with adverse health outcomes have also been found in studies using medical records to ascertain health outcomes^5,30^ and in studies using court-documented cases of abuse,^64^ suggesting that associations of ACEs with health outcomes are robust to recall bias.

### Limitations

Our study has several limitations. Players who reported more vs fewer concussion symptoms in wave 1 were less likely to participate in wave 2. This and other differences in participation may have caused bias. Moreover, the second wave of data collection had modest participation, although dropout would need to be associated with both our outcomes and ACE exposure to have biased our results. ACEs and concussion history were retrospectively self-reported and may be subject to recall bias. Unmeasured factors, including genetic liability to mental illness, may be associated with both ACEs and neuropsychiatric outcomes^65^; thus, our estimates may include residual confounding. Players in good health may be less likely than players in poor health to recall childhood adversities they experienced. Finally, the prevalence of positive dementia screening results was high compared with the US population in this age range.^66^ Our sample may have elevated prevalence of traumatic encephalopathy syndrome, or publicity associated with chronic traumatic encephalopathy may have made former players more aware of cognitive change compared with the general population. In addition, former football players have a high prevalence of (often treatable) comorbidities that can affect cognition.^67,68^

## Conclusions

The finding of this study suggest that ACEs may constitute a risk factor for a positive dementia screening result, poor cognition-related quality of life, and concussion symptoms among former professional US football players. Furthermore, ACEs may be a prospective indicator of players who are at high risk for concussion. Former players and their clinicians should consider treatment of psychological trauma in addition to treatment of physical injury to improve neurobehavioral outcomes. Emotion regulation strategies, narration of trauma memory, anxiety and stress management, interpersonal skills training, mindfulness, and meditation are current best practices for treatment of trauma.^69^ In former players with a history of family dysfunction, treatment appropriate to this history may improve cognitive and psychological functioning.^70,71^

## Notes

Supplement. **eTable 1.** Correlation of Childhood Adversities, Football Players Health Study, 2018-2021 (N = 1755) **eTable 2.** Correlation of Neuropsychiatric Outcomes, Football Players Health Study, 2018-2021 (N = 1755) **eTable 3.** Adverse Childhood Experiences (ACEs) and 6 Neuropsychiatric Health Outcomes, Football Players Health Study, 2018-2021 (N = 1755)
