# Supplementary material for: Association of Adverse Childhood Experiences With Poor Neuropsychiatric Health and Dementia Among Former Professional US Football Players
Source: JAMA Netw Open. 2022 Mar 22;5(3):e223299. doi: 10.1001/jamanetworkopen.2022.3299 (PMC8941347; doi:10.1001/jamanetworkopen.2022.3299)
Supplement: Supplement. — eTable 1. Correlation of Childhood Adversities, Football Players Health Study, 2018-2021 (N = 1755) eTable 2. Correlation of Neuropsychiatric Outcomes, Football Players Health Study, 2018-2021 (N = 1755) eTable 3. Adverse Childhood Experiences (ACEs) and 6 Neuropsychiatric Health Outcomes, Football Players Health Study, 2018-2021 (N = 1755) [file jamanetwopen-e223299-s001.pdf]

Supplementary Online Content

Roberts AL, Zafonte R, Chibnik LB, et al. Association of adverse childhood experiences with poor neuropsychiatric health and dementia among former professional US football players. *JAMA Netw Open*. 2022;5(3):e223299. doi:10.1001/jamanetworkopen.2022.3299

eTable 1. Correlation of Childhood Adversities, Football Players Health Study, 2018-2021 (N = 1755)

eTable 2. Correlation of Neuropsychiatric Outcomes, Football Players Health Study, 2018-2021 (N = 1755)

eTable 3. Adverse Childhood Experiences (ACEs) and 6 Neuropsychiatric Health Outcomes, Football Players Health Study, 2018-2021 (N = 1755)

This supplementary material has been provided by the authors to give readers additional information about their work.

eTable 1. Correlation of Childhood Adversities, Football Players Health Study, 2018-2021 (N = 1755)

	Prevalence	Emotional abuse	Physical abuse	Sexual abuse	Emotional neglect	Physical neglect	Divorce	Intimate partner violence	Substance user	Mental illness	Prison
	N (%)										
Emotional abuse	337 (19.3)	1.00	0.59	0.13	0.34	0.22	0.23	0.35	0.25	0.22	0.19
Physical abuse	363 (20.7)		1.00	0.14	0.28	0.24	0.17	0.35	0.23	0.17	0.16
Sexual abuse	111 (6.3)			1.00	0.11	0.15	0.13	0.12	0.13	0.12	0.09
Emotional neglect	117 (6.7)				1.00	0.31	0.13	0.21	0.19	0.16	0.16
Physical neglect	82 (4.7)					1.00	0.19	0.28	0.22	0.20	0.17
Divorce/separation	527 (30.2)						1.00	0.26	0.27	0.16	0.21
Intimate partner violence to mother	147 (8.4)							1.00	0.27	0.21	0.21
Household member substance user	406 (23.2)								1.00	0.23	0.23
Household member mental illness/suicidal	169 (9.7)									1.00	0.15
Household member imprisoned	134 (7.7)										1.00

All correlations significant at *P* = .000

© 2022 Roberts AL et al. *JAMA Network Open*.

eTable 2. Correlation of Neuropsychiatric Outcomes, Football Players Health Study, 2018-2021 (N = 1755)

		Positive dementia screen	Poor cognition-related QoL	Probable depression	Probable anxiety	Pain severity	Pain interference
	N cases (%)	Pearson correlation coefficient					
Positive dementia screen	752 (43.1)	1.00	0.67	0.45	0.36	0.28	0.39
Poor cognition-related QoL	656 (37.7)		1.00	0.50	0.37	0.29	0.38
Probable moderate or severe depression	291 (16.6)			1.00	0.59	0.31	0.47
Probable moderate or severe anxiety	194 (11.1)				1.00	0.29	0.41
High pain severity	556 (32.1)					1.00	0.57
High pain interference	421 (24.2)						1.00

All correlations significant at *P* < .0001

© 2022 Roberts AL et al. *JAMA Network Open*.

eTable 3. Adverse Childhood Experiences (ACEs) and 6 Neuropsychiatric Health Outcomes, Football Players Health Study, 2018-2021 (N = 1755)

	**Adverse childhood experiences (ACEs)**					
	**None**	**1**	**2**	**3**	**4 or more**	**Test of trend**
	n=767	n=400	n=245	n=135	n=208	
	Risk ratio (95% confidence interval)					*P* value
Outcome: Positive dementia screen						
Base model adjusted for age, race, and childhood SES	1.0 [Reference]	1.10 (0.91-1.33)	1.12 (0.92-1.39)	**1.27 (1.00-1.59)**	**1.48 (1.22-1.79)**	<.001
Further adjusted for concussions	1.0 [Reference]	1.04 (0.88-1.24)	1.09 (0.89-1.33)	1.14 (0.93-1.40)	**1.32 (1.10-1.58)**	.004
Outcome: Poor cognition-related quality of life						
Base model adjusted for age, race, and childhood SES	1.0 [Reference]	1.14 (0.92-1.41)	**1.29 (1.03-1.62)**	**1.40 (1.09-1.80)**	**1.65 (1.33-2.04)**	<.001
Further adjusted for concussions	1.0 [Reference]	1.08 (0.89-1.32)	**1.25 (1.02-1.55)**	**1.26 (1.00-1.59)**	**1.47 (1.21-1.79)**	<.001
Outcome: Probable depression						
Base model adjusted for age, race, and childhood SES	1.0 [Reference]	1.27 (0.89-1.82)	0.78 (0.48-1.26)	**1.65 (1.06-2.55)**	**1.62 (1.09-2.39)**	.01
Further adjusted for concussions	1.0 [Reference]	1.20 (0.85-1.69)	0.77 (0.49-1.22)	1.38 (0.92-2.06)	1.35 (0.93-1.97)	.13
Outcome: Probable anxiety						
Base model adjusted for age, race, and childhood SES	1.0 [Reference]	1.21 (0.79-1.86)	0.82 (0.48-1.40)	1.16 (0.63-2.12)	1.46 (0.89-2.39)	.22
Further adjusted for concussions	1.0 [Reference]	1.15 (0.77-1.72)	0.85 (0.50-1.40)	0.96 (0.54-1.69)	1.21 (0.74-1.96)	.71
Outcome: Moderate or severe pain intensity						
Base model adjusted for age, race, and childhood SES	1.0 [Reference]	1.00 (0.79-1.27)	1.05 (0.80-1.37)	**1.46 (1.12-1.91)**	**1.48 (1.16-1.90)**	.001
Further adjusted for concussions	1.0 [Reference]	0.97 (0.77-1.22)	1.03 (0.79-1.33)	**1.37 (1.06-1.77)**	**1.37 (1.07-1.74)**	.003
Outcome: Moderate or severe pain interference						
Base model adjusted for age, race, and childhood SES	1.0 [Reference]	1.14 (0.85-1.54)	1.34 (0.98-1.85)	**1.75 (1.25-2.44)**	**1.74 (1.27-2.40)**	<.001
Further adjusted for concussions	1.0 [Reference]	1.11 (0.84-1.48)	1.33 (0.98-1.80)	**1.60 (1.16-2.21)**	**1.56 (1.15-2.11)**	.001

Notes: Concussions are concussion symptoms at the time of football injury. Poor cognition-related quality of life indicates “moderate” or “severe” impairment on the Neuro-QOL. Bolded estimates are statistically significant.

© 2022 Roberts AL et al. *JAMA Network Open*.
